# Intraosseous Ganglion Protruding Into the Spinoglenoid Notch With Suprascapular Nerve Entrapment: A Case Report

**DOI:** 10.7759/cureus.74863

**Published:** 2024-11-30

**Authors:** Gaku Matsuzawa, Taku Hatta, Shigeyuki Asano, Masaki Takahashi, Toshitake Aizawa

**Affiliations:** 1 Department of Orthopaedic Surgery, Iwaki City Medical Center, Iwaki, JPN; 2 Department of Orthopaedic Surgery, Tome Citizen Hospital, Tome, JPN; 3 Department of Orthopaedic Surgery, Joint Surgery Sports Clinic Ishinomaki, Ishinomaki, JPN; 4 Pathology Center, Iwaki City Medical Center, Iwaki, JPN

**Keywords:** entrapment, ganglion, intraosseous, spinoglenoid notch, suprascapular nerve

## Abstract

Suprascapular nerve entrapment caused by intraosseous cystic lesions is a rare condition. We present the case of a 49-year-old man with right shoulder numbness, slight infraspinatus (ISP) weakness, and shoulder pain. He underwent open surgery and arthroscopic evaluation. The cystic lesion, histologically diagnosed as a ganglion, was resected, and the bony defect was filled with artificial bone. Immediately after the operation, the shoulder numbness and muscle weakness resolved. At the one-year follow-up, the patient’s right shoulder pain disappeared, and he had excellent clinical results. An intraosseous cyst protruding into the spinoglenoid notch, causing suprascapular nerve entrapment, might elicit neurological symptoms and glenoid fracture. However, the treatment and clinical course of this condition are still unknown. This case suggests that patients treated with tumor resection and bone grafting to the bony defect in this abnormality may have favorable outcomes.

## Introduction

Shoulder pain is a common condition; however, the symptoms caused by nerve entrapment are relatively uncommon. The suprascapular nerve is a mixed sensory and mainly supraspinatus (SSP) and infraspinatus (ISP) motor nerve originating from the brachial plexus [1,2]. Because suprascapular nerve entrapment is a rare condition, typically presenting as nonspecific shoulder pain and weakness, it can be easily overlooked in the differential diagnosis of shoulder dysfunction. Suprascapular nerve entrapment can be caused by impingement between the ligamentous structures on the notch-like structures and the neighboring mass region [1,3,4]. Suprascapular nerve entrapment by a mass such as a paralabral ganglion cyst has been described in some studies [3,5-7]. In contrast, suprascapular nerve entrapment in the spinoglenoid notch caused by an intraosseous ganglion of the glenoid is an extremely rare condition, and only a few studies have reported this abnormality [8,9]. Furthermore, its treatment method and clinical course are still unknown. To the best of our knowledge, no studies have been published about this region regarding tumor resection and prevention of the subsequent glenoid fracture with images and arthroscopic findings.

This is a report of a case of intraosseous ganglia behind the thin glenoid wall that presented as suprascapular nerve entrapment at the spinoglenoid notch. The patient provided informed consent to the surgical procedure and publication of the report.

## Case presentation

**Figure 1:**
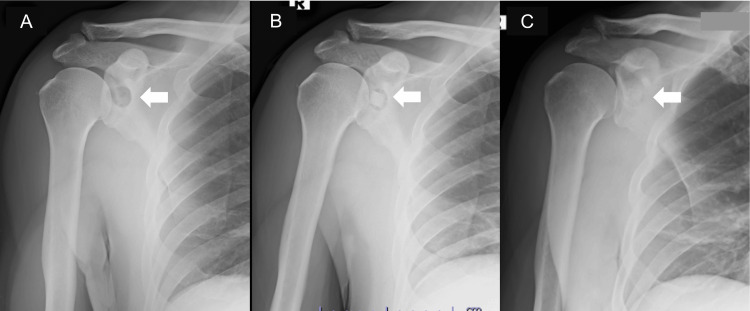
Right shoulder anteroposterior X-ray images (A) The preoperative image illustrates a radiolucency area with a well-defined margin at the midportion of the glenoid. (B) The postoperative image indicates beta-tricalcium phosphate (β-TCP) at the radiolucent region. (C) The region was almost filled with artificial and replacement bone in the six-month follow-up. The white arrow denotes an intraosseous cystic lesion.

**Figure 2:**
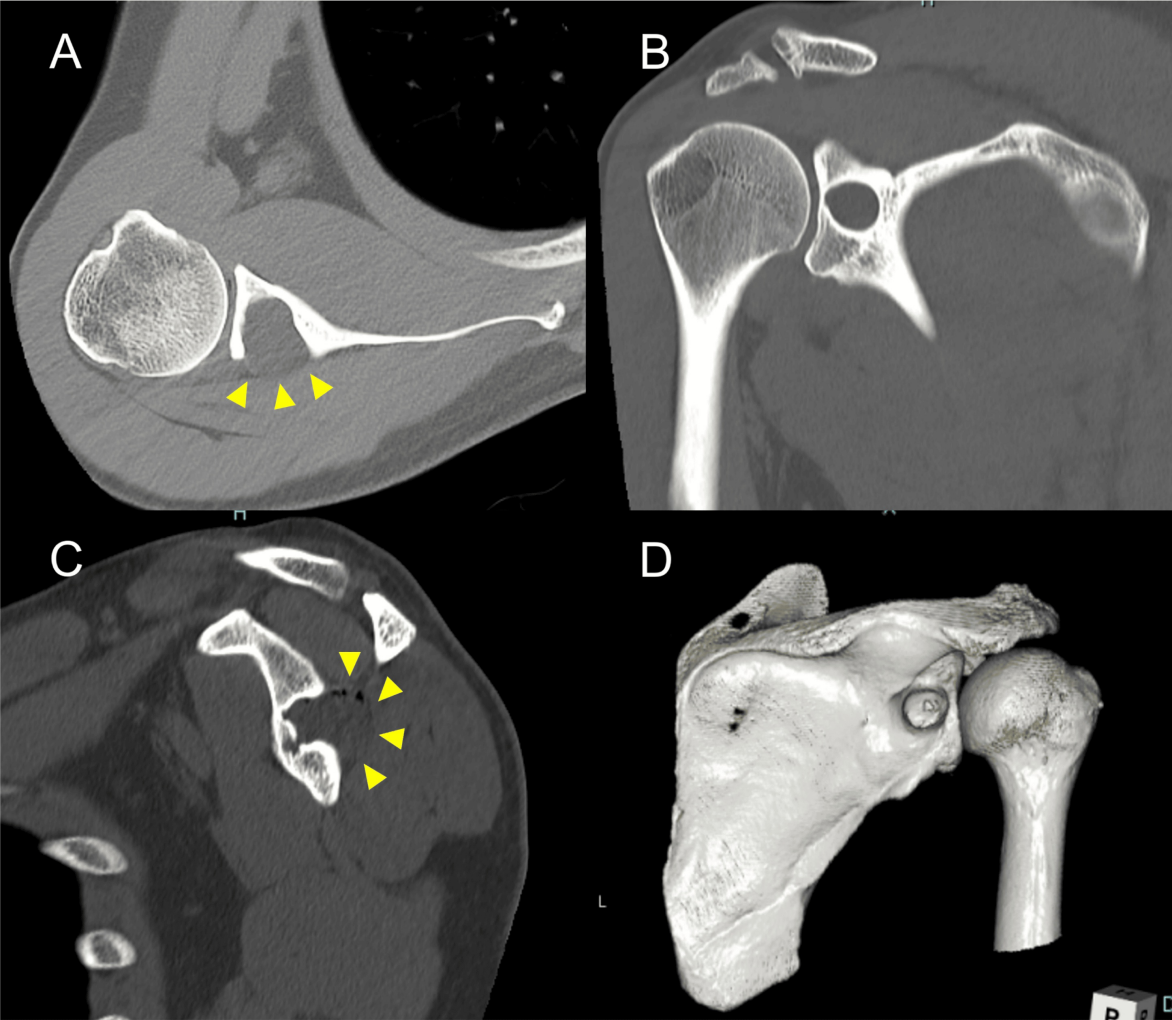
Preoperative right shoulder computed tomography (CT) images The preoperative (A) axial, (B) coronal, (C) sagittal, and (D) posterior three-dimensional (3D) views of CTs show a bony defect area at the mid-portion of the glenoid containing a cystic lesion (triangle heads).

**Figure 3:**
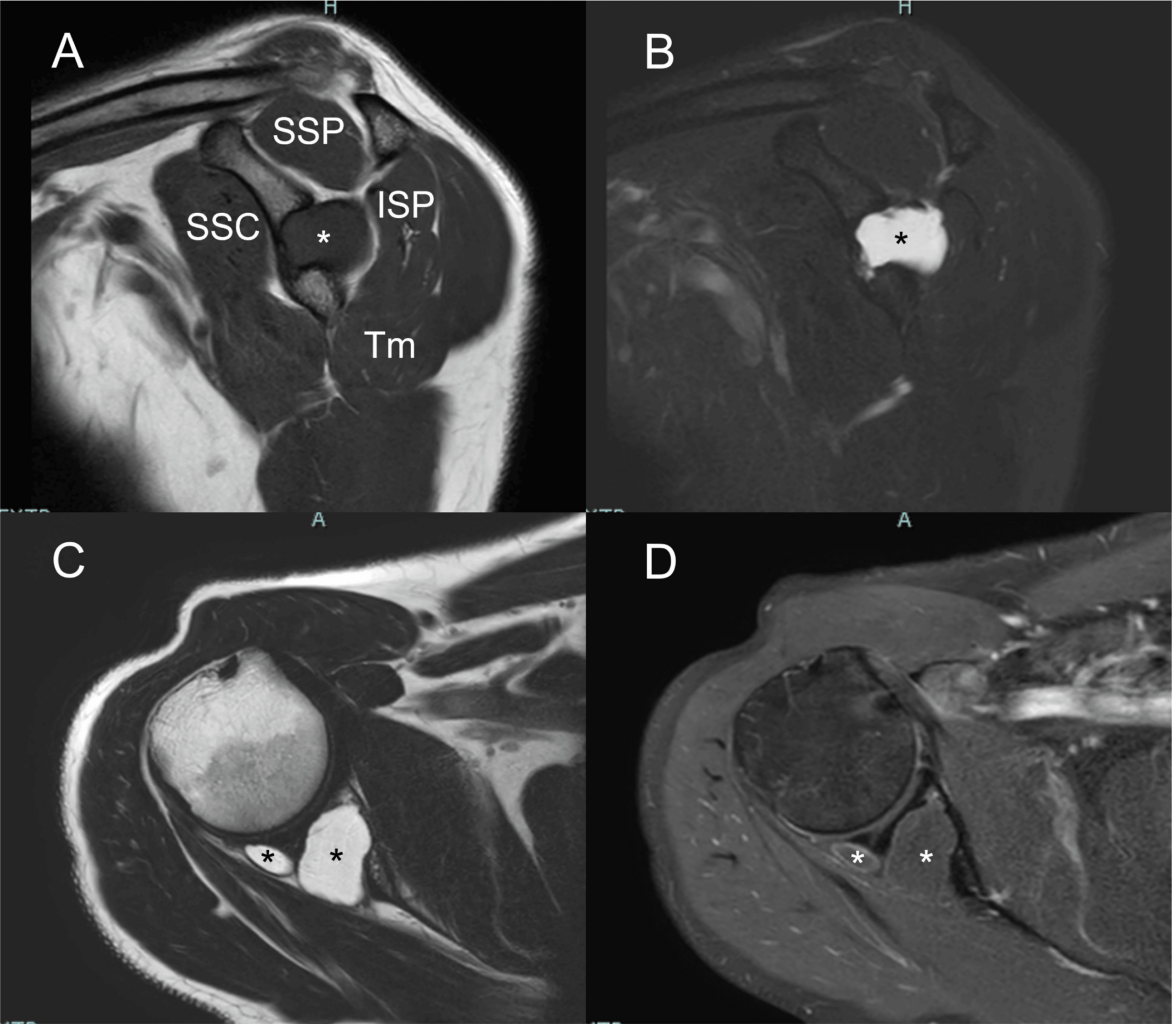
Preoperative right shoulder magnetic resonance imaging The preoperative (A) T1- and (B) T2-weighted fat suppression images in the sagittal plane and (C) T2-weighted and (D) gadolinium-enhanced T1-weighted fat suppression images in the axial plane demonstrating an intraosseous cystic lesion with enhanced margins extended to the spinoglenoid notch. *, partly multi-nodule cystic lesion; SSP, supraspinatus; ISP, infraspinatus; Tm, teres minor; SSC, subscapularis

A 49-year-old man (height, 172 cm; weight, 86 kg; right-hand dominant; truck driver), who had endured a chronic dull ache and numbness in his right shoulder for one year without any trigger, had felt his sensory impairment exacerbated for three months. He had no history of trauma or any other initial adverse events to his shoulders. The pain worsened when he was lying on his right side or bore a heavy burden on his right arm. His medical history was unremarkable. He had no history of smoking. His symptoms did not improve with physical therapy and medications such as non-steroidal anti-inflammatory drugs (NSAIDs) and α2δ ligands. Physical examination revealed mild numbness in the posterior part of the patient’s right shoulder at which sensory impairment had exacerbated for a few months, deep and non-localized pain that aggravated by applying pressure to the right shoulder, full elevation and external rotation range of motion (ROM), and very slight external rotation weakness (with 5 minus in the manual muscle test [MMT]) without external rotation rag sign. No muscle atrophy was observed. Plain anteroposterior radiography of the right shoulder showed a round-shaped area of radiolucency with a well-defined margin and sclerotic rim in the subchondral area at the midportion of the glenoid (Figure 1A). Computed tomography (CT) revealed a cystic lesion with sclerotic margins without a posterior osseous wall (Figures 2A-2D). Magnetic resonance imaging (MRI) showed a partly multi-noduled elliptical-shaped mass communicating both in and out of the osteolytic area, low intensity in T1-weighted images, high intensity in T2-weighted images, and low intensity with contrasted margin in gadolinium-enhanced T1-weighted images. It was located in the posterior portion of the glenoid and extended to the spinoglenoid notch. The main lesion of the mass measured 24 × 18 × 27 mm in diameter (Figures 3A-3D).

**Figure 4:**
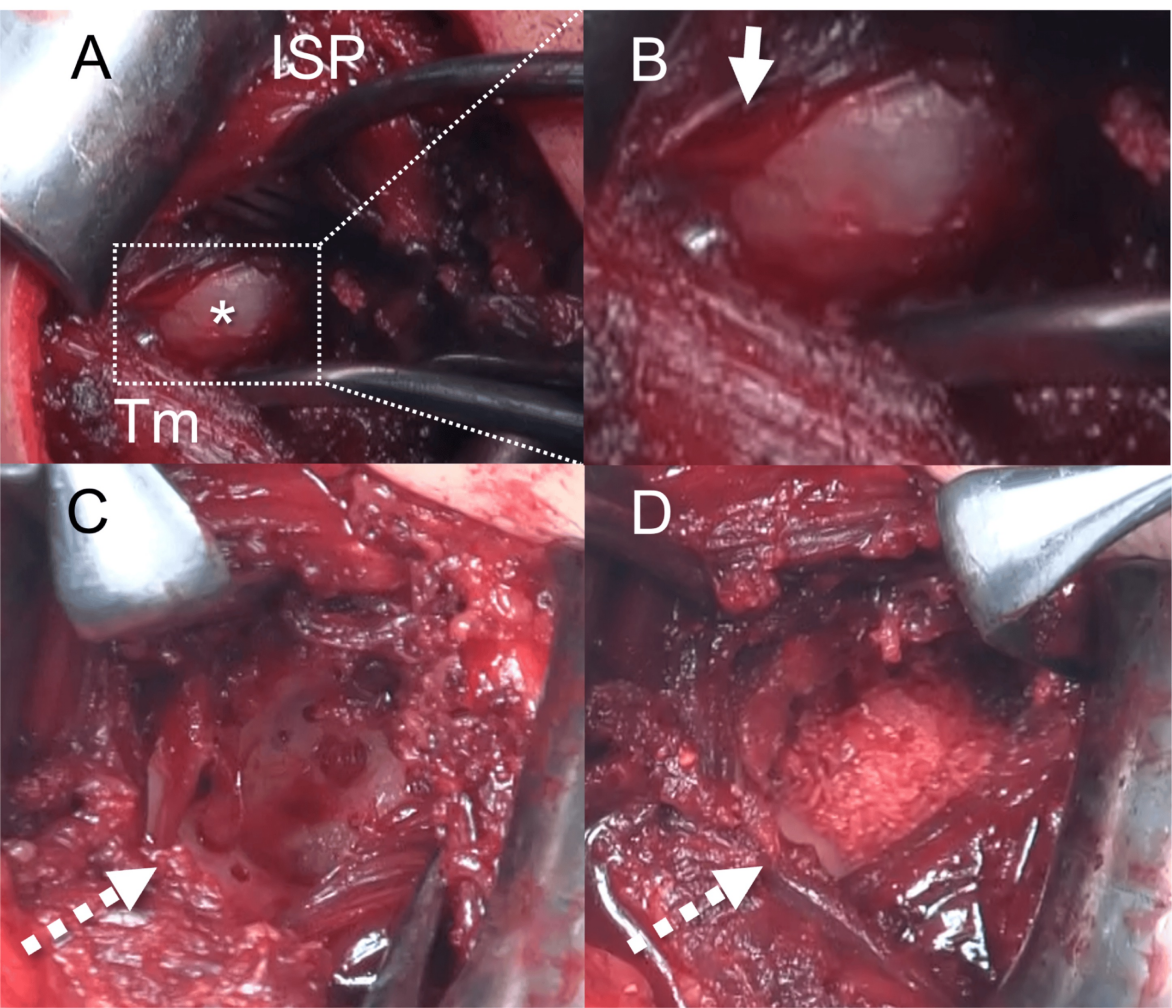
Surgical findings (A) The cystic lesion (dotted area) detected between the infraspinatus and teres minor. (B) The tumor compressing the suprascapular nerve (arrow). The dotted arrow indicates post-curetted bone defect (C) filled with β-TCP (D). *, cystic lesion; ISP, infraspinatus; Tm, teres minor; β-TCP, beta-tricalcium phosphate

**Figure 5:**
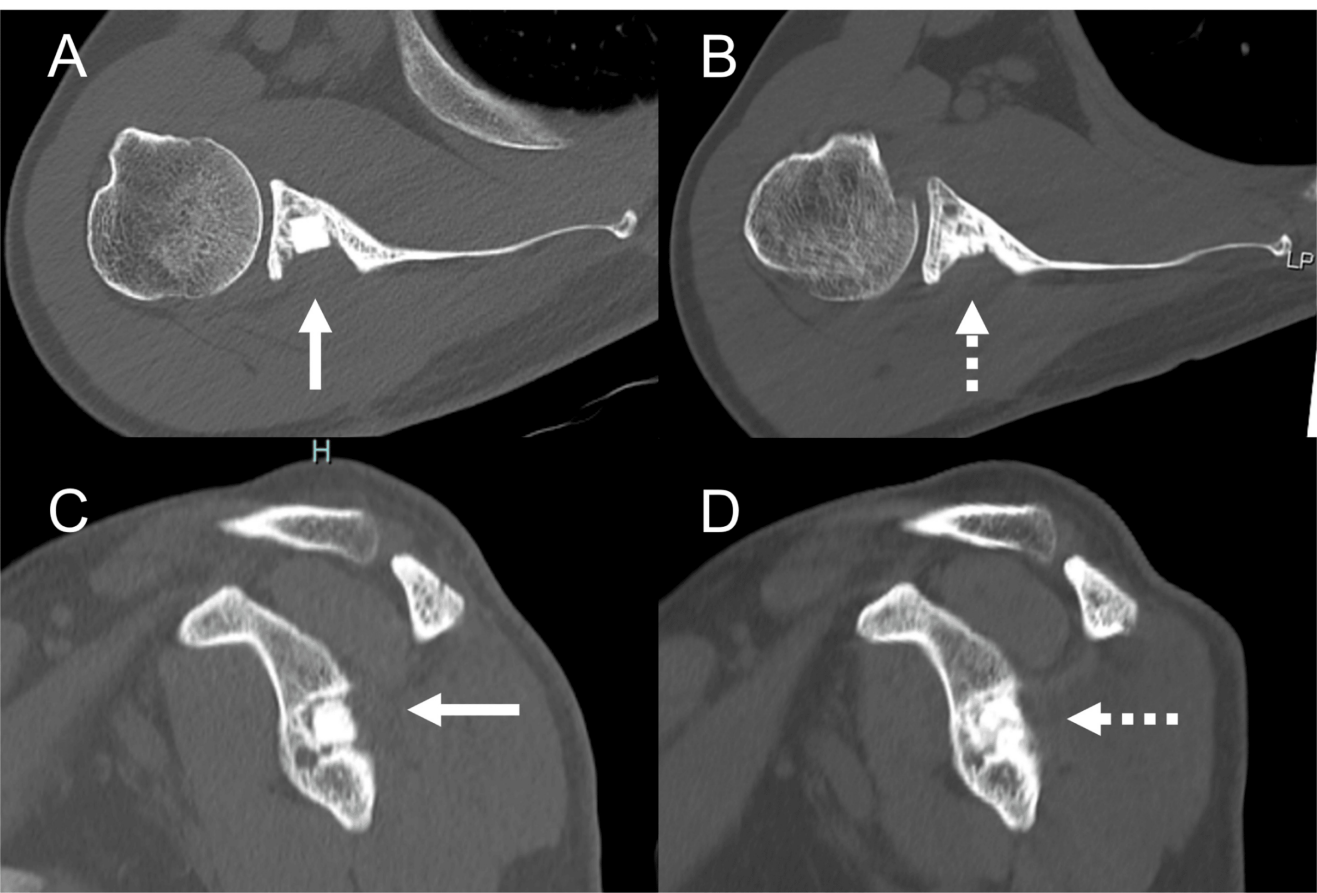
Postoperative right shoulder CT images The one-week postoperative (A) axial and (C) coronal plane images illustrate a bony defect containing beta-tricalcium phosphate (β-TCP) (arrow). (B,D) The defect was filled with β-TCP and replaced bone at the six-month follow-up (dotted arrow).

The preoperative American Shoulder and Elbow Surgeons (ASES) score and Constant-Murley score (CMS) were 72 and 82 points, respectively. The cause of the patient’s symptoms might have been nerve entrapment caused by the cystic lesion and the impending glenoid fracture in relation to the bone defect. Although needle aspiration may be an effective treatment for nerve entrapment caused by cystic lesions, it may not be effective in treating impending glenoid fractures. Because of the chronicity of the symptoms, the patient chose surgery without needle aspiration after receiving a sufficient explanation of his condition. The operation with the Brodsky approach under general anesthesia in the left lateral position was performed. The operation time was 141 minutes, and the blood loss was 163 mL. The tumor was directly visualized between the ISP and teres minor (Tm), with the suprascapular nerve on the craniomedial surface of the tumor (Figures 4A, 4B). The tumor was resected with its septum until the bone surface was exposed (Figure 4C). The bone defects were filled with beta-tricalcium phosphate (β-TCP) (Figures 1B, 4D, 5A, 5C).

**Figure 6:**
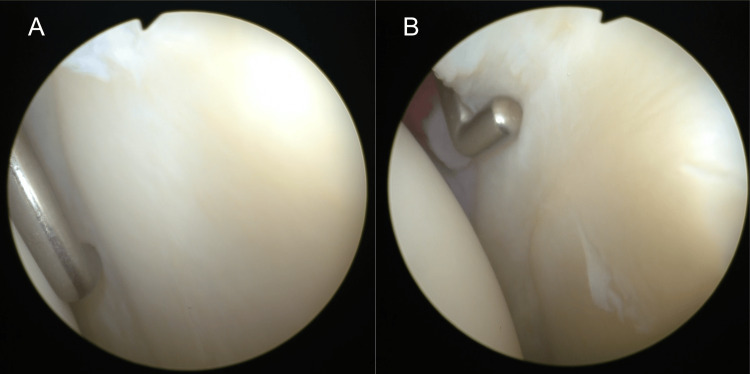
Arthroscopic right shoulder results Arthroscopic posterior view of the glenohumeral joint from the anterior portal, not revealing a connection tract between the glenohumeral joint and intraosseous cyst (A) without the SLAP lesion (B). SLAP, superior labrum anterior-posterior

In the arthroscopic findings, no communication was noted between the cystic lesion and the glenohumeral joint without the superior labrum anterior-posterior (SLAP) lesion, which was the same as the radiological findings (Figures 6A, 6B).

**Figure 7:**
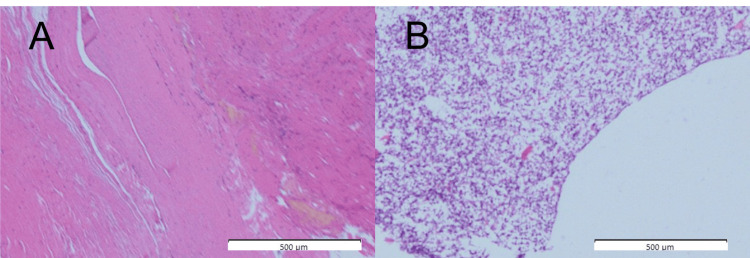
Histological findings The resected tumor composed of fibrous connective tissue (A) and myxoid contents (B), as revealed by hematoxylin and eosin staining; thus, this cystic lesion was diagnosed as a ganglion cyst.

Histological examination revealed that the septa of the tumor were composed of connective tissue and mucinous contents. These findings were consistent with a ganglion (Figures 7A, 7B).

**Figure 8:**
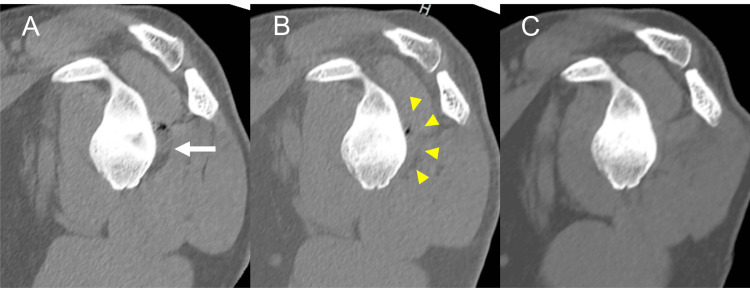
Radiological course of the cystic lesion protruding into the spinoglenoid notch The cystic lesion (arrow) on the preoperative CT (A) was resected, but a small space occupied by a suspected hematoma remained (triangle heads) by operation (B). (C) No lesion recurrence was reported at the six-month follow-up.

**Figure 9:**
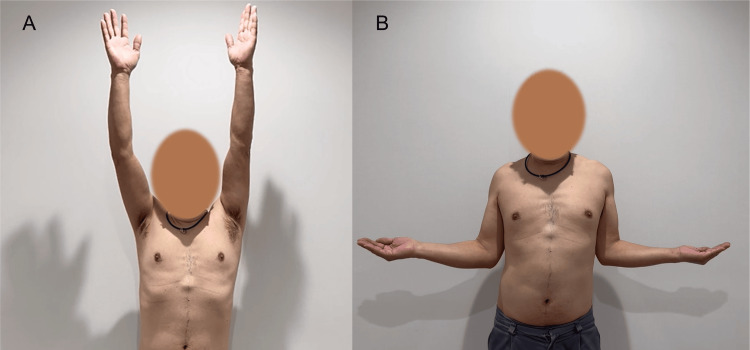
Shoulder function at one-year follow-up Excellent postoperative shoulder function, including active elevation (A) and external rotation (B).

Gentle stretching and ROM exercises of the shoulder began the day after surgery with a simple sling for one week. At six weeks, the patient progressed to full strengthening exercises without restrictions. Immediately after the operation, the patient’s shoulder numbness and muscle weakness resolved. However, the deep right shoulder pain remained to some extent when the patient was lying on his right side. At the six-month follow-up, the patient’s right shoulder pain disappeared, and he had no functional deficits without recurrence of the mass lesion (Figures 8A-8C). The bony defect was filled with β-TCP and replaced bone (Figures 1C, 5B, 5D). The symptoms went away, and the patient had excellent shoulder function with 98 and 96 points of ASES and CMS with full elevation and external rotation, respectively (Figures 9A, 9B), in the one-year follow-up.

## Discussion

We experienced a case of the intraosseous ganglia in the scapula protruding into the spinoglenoid notch, unlike soft tissue ganglion, which is quite rare. In this study, numbness and very slight muscle weakness were resolved immediately after the operation; however, the weak pain remained while lying on his right side. Concerning these clinical courses, his numbness and muscle weakness were caused by the suprascapular nerve compression; however, shoulder pain was caused by nerve compression and/or glenoid bone stress, and an impending fracture was possible.

Treatment might be required if the ganglion protrudes into the spinoglenoid notch and compresses the adjacent nerve. A paralabral cyst associated with the SLAP lesion making a one-way valve could be one of the causes of suprascapular nerve compression at the spinoglenoid notch [10-12]. Moreover, in the case of an intraosseous ganglion occurring just behind the glenoid, treatment for a consecutive glenoid fracture should be considered. For paralabral cysts, because there is a connection between the glenohumeral joint and the cystic lesion, the lesion can be removed from the articular side via arthroscopic surgery [10-13]. However, no link has been reported between the glenohumeral joint and intraosseous ganglion, unlike in paralabral cysts [8,9,14,15]. Therefore, it may be difficult to adequately remove intraosseous cystic lesions using an arthroscopic approach. Furthermore, even in the case of a paralabral cyst with neural compression, open surgery can be recommended for cyst excision if the cyst cannot be seen through the SLAP lesion or if no SLAP lesion is present [13]. In the present study, as reported previously, no link appeared between the ganglion and glenohumeral joint; therefore, an open surgical procedure was performed to completely remove the ganglion. Electromyography (EMG) might be performed for the diagnosis, but because there might be many branches of the suprascapular nerve, the EMG-based diagnosis of nerve entrapment might be difficult [13]. Moreover, EMG may be useful for comparing muscle functions before and after surgery. We did not evaluate muscle function with EMG because we assessed the SSP and ISP strength, and strength was evaluated as intact SSP and slight weakness of ISP as 5 minus in MMT. The patient’s symptoms were caused by suprascapular nerve compression by the ganglion, and additional EMG findings did not validate the change to our treatment strategy. Needle aspiration is a feasible treatment option for cystic lesions protruding into the spinoglenoid notch; however, some problems remain with this strategy. In the case of a paralabral cyst treated by needle aspiration, >60% of patients were satisfied with their outcome, but some had recurrent cyst formation, according to a previous study [5]. Conversely, an intraosseous bone cyst might be difficult to reduce completely using only needle aspiration [9]; access would prove challenging because a needle inserted from the body surface would be unable to evenly reach a cyst that fills an intricately shaped bone defect; in addition, such cysts occasionally have multiple nodules, as in the current case. A study reported being treated with arthroscopic aspiration without bone grafting in the post-aspiration bone defect for the intraosseous ganglion in the glenoid neck, which compressed the suprascapular nerve at the “supraglenoid” notch [16]. The postoperative glenoid fracture did not occur in this report; however, the lesion was in the upper part of the glenoid fossa, and the same treatment would not be effective for lesions in glenoid areas that might be subject to heavy load. The other case of suprascapular nerve entrapment caused by an intraosseous ganglion [8,9] was treated by the curettage of the cystic lesion without bone grafting; however, the subchondral bone remained to a certain extent, and the patient’s shoulder pain disappeared immediately after the operation, which was different from our latest case. Furthermore, in cystic lesions that cause bone defects in the glenoid neck, a risk of glenoid fracture is possibly due to stress on the glenoid joint [14,15,17]. However, we do not know the cutoff thickness of the remaining subchondral bone for the treatment to avoid subsequent glenoid fracture, and without surgical resection of the cystic lesions might cause the cyst to progress and penetrate the glenoid wall, possibly causing cartilage destruction and eventual osteoarthritis. The intraosseous ganglion with impending fracture of the thinning glenoid could be treated by the ganglion curettage and packing the cavity with autologous bone and/or artificial bone chips [14,15]. Therefore, we treated the thinning glenoid with pain caused by the osteolytic cystic lesion not only to relieve nerve entrapment but also to prevent the possibility of glenoid fractures by sufficient bone grafting in the glenoid bone defect. Regarding the size of the lesion and the possibility of both nerve entrapment and impending glenoid fracture in the current case, neither treatment through puncture aspiration of the cystic lesion nor treatment of arthroscopic curettage was adopted; instead, open surgery with the Broadsky approach was performed to curettage the cystic lesion, release a nerve entrapment, and fill the artificial bone graft in the bony defect.

Whether the cause of the shoulder pain was due to stress from the thinning of the glenoid or due to nerve compression by a ganglion, in our current case, was unclear. Therefore, operative findings and postoperative clinical courses were helpful. In this case, the disappearance of shoulder numbness and weakness of the ISP immediately after the surgery was strongly suspected to be related to nerve entrapment by the cystic lesions, and the remaining shoulder pain lying on the patient’s right side immediately after the surgery was strongly suspected to be related to the stress to the thinning glenoid. Furthermore, weeks after the surgery, the patient’s shoulder pain completely disappeared, which might be due to the bone graft filling the defect behind the glenoid being replaced by bone and stabilizing the glenoid.

Even though the procedure described herein was performed under very limited conditions, our findings suggest that the proposed treatment method is a good option for the type of abnormality, such as cases reported in this study.

## Conclusions

This report describes a treatment option for a rare case of an intraosseous ganglion that caused glenoid fossa thinning and suprascapular nerve compression at the spinoglenoid notch, resulting in shoulder pain, numbness, and mild muscle weakness. Open surgery was performed for ganglion resection and bone grafting to the bone defect behind the glenoid. Postoperatively, numbness and muscle strength improved quickly; however, shoulder pain remained. The shoulder pain disappeared at the six-month follow-up, and the symptoms did not recur at the one-year follow-up. In cases of the intraosseous ganglion of the glenoid protruding into the spinoglenoid notch, sufficient ganglion resection and bone grafting via open surgery are important to relieve nerve compression and avoid subsequent glenoid fractures.
